# Buffalo State Asylum for the Insane

**Published:** 1872-09

**Authors:** 


### Buffalo State Asylum for the Insane. Laying of the Corner Stone.

The corner stone of the Buffalo Insane Asylum was laid Sept. 18th with appropriate Masonic honors by M. W. Christopher, G. Fox. Grand Master of the State of New York, in the presence of a large number of Masons of the city and vicinity. Governor Hoffman was present on the occasien and a large number of invited guests, among whom we noticed the Mayor and several members of the Common Council, the clergy and members of various organizations. The exercises were opened with a prayer by the Rev. Dr. Lord following which was an appropriate address, by this Excellency Gov. Hoffman.

At the conclusion of the Governors address Prof. James P. White, M. D., President of the Board of Managers in a few well chosen remarks invited the Masons to lay the corner stone which was accordingly done with appropriate honors.

The Oration of Hon. James O. Putnam which was to have been delivered on the occasion was omitted on account of the ill health of the orator and the inclemency of the weather. We had, however, the pleasure of reading the speech as published in the daily papers and were much pleased and instructed in its perusal. The exercises were agreably varied by music by the regimental bands which together with the military under command of Generals Howard and Rogers acted as an escort to Gov. Hoffman and the masonic lodges.

The work on the Insane Asylum has progressed quite rapidly under the direction of the efficient board of Managers.

The foundation for the central building and two of the wings has already been laid and the walls erected to a considerable extent. In a short time Buffalo will have an Insane Asylum, which for accommodation of the insane and beauty of situation will rank second to none in the United States.

The citizens of Western New York should acknowledge their indebtedness to Prof. James P. White of Buffalo as chiefly instrumental in the origin and location of this great State charity.

